# Pre-diagnostic leukocyte mitochondrial DNA copy number and risk of lung cancer

**DOI:** 10.18632/oncotarget.8426

**Published:** 2016-03-27

**Authors:** Shasha Meng, Immaculata De Vivo, Liming Liang, Zhibin Hu, David C. Christiani, Edward Giovannucci, Jiali Han

**Affiliations:** ^1^ Department of Epidemiology, Harvard School of Public Health, Boston, Massachusetts, USA; ^2^ Channing Division of Network Medicine, Brigham and Women's Hospital and Harvard Medical School, Boston, Massachusetts, USA; ^3^ Department of Epidemiology and Biostatistics, School of Public Health, Nanjing Medical University, Nanjing, China; ^4^ Department of Environmental Health, Harvard University School of Public Health, Boston, Massachusetts, USA; ^5^ Department of Medicine, Harvard Medical School, Boston, Massachusetts, USA; ^6^ Department of Nutrition, Harvard University School of Public Health, Boston, Massachusetts, USA; ^7^ Department of Epidemiology, Richard M. Fairbanks School of Public Health, Indiana University, Indianapolis, Indiana, USA; ^8^ Melvin and Bren Simon Cancer Center Indiana University, Indianapolis, Indiana, USA

**Keywords:** mitochondrial DNA copy number, lung cancer risk, oxidative stress, prospective cohort, case-control study

## Abstract

We prospectively investigated the relationship between mtCN and the risk of lung cancer in 463 case-control pairs from two prospective cohort studies, the Nurses’ Health Study (NHS) and the Health Professionals Follow-Up Study (HPFS). The adjusted least-squares means of log-transformed mtCN (log_mtCN) by smoking status were estimated by generalized linear models. Multivariable conditional logistic regression model adjusting for confounders was used to obtain the odds ratios (ORs) and 95% confidence intervals (CIs) for the association between log_mtCN and lung cancer risk. The adjusted least-squares mean of log_mtCN in heavy smokers was significantly lower than that in never smokers (*P* = 0.05). Compared to the high log_mtCN group, the risk of lung cancer was 1.29 (95% CI = 0.89–1.87) for the median group, and 1.11 (95% CI = 0.75–1.64) for the low group. Among current smokers, compared to participants with high levels of log_mtCN, those with median levels had a significantly higher risk of lung cancer (OR = 2.09; 95% CI = 1.12–3.90), but not those with low levels (OR = 1.37; 95% CI = 0.75–2.48). Further studies are warranted to confirm these findings.

## INTRODUCTION

Mitochondria are organelles in the cytoplasm within a eukaryotic cell. Their main functions include energy metabolism, free radical production, calcium homeostasis, and apoptosis [1]. Mitochondrial DNA (mtDNA) is closely located to the source of reactive oxidative stress (ROS) production and is extremely susceptible to oxidative or other genotoxic damage due to the absence of protective histones, the lack of introns, and scarcity of efficient DNA repair mechanisms. As a result, mtDNA acquires mutations at a much higher rate (10– to 200– fold) than nuclear DNA) [2, 3]. Consequently, the mitochondrial DNA copy number (mtCN) may either increase or decrease under the combined effect of mitochondrial and nuclear DNA mutations and these changes are to some degree tumor specific [3]. For instance, somatic point mutations close to the replication region in the D-loop, the non-coding region in mtDNA that is the major regulatory site for mitochondrial genome replication and transcription, were significantly associated with reduced mtDNA content in hepatocellular carcinoma, invasive breast cancer, and Ewing's sarcoma (EWS) [4–6]. It is hypothesized that D-loop alterations might modify the binding affinities of some regulators encoded by nuclear DNA (e.g., mitochondrial transcription factor A, or TFAM) on the mtDNA replication site [7]. Furthermore, the regulatory role of nuclear DNA in encoding trans-acting factors responsible for mitochondrial biogenesis and mtDNA maintenance (e.g., mitochondrial single-strand DNA binding protein or mtSSB) was found to be disrupted in certain cancers [8]. In addition, nuclear alterations in some key players such as p53 and mtDNA polymerase γ (POLG) are also present with mtDNA content changes in several cancer types [9, 10].

It has been estimated that cigarette smoking is responsible for up to 90% of lung cancer development. Results from both laboratory experiments and human studies suggest that cigarette smoking can lead to increased oxidative damage. Generally, smokers show higher levels of plasma lipid peroxidation, mtDNA content and urinary secretion of 8-OHdG compared to nonsmokers [11, 12]. Given that mtDNA copy number (mtCN) in leukocytes is highly correlated with oxidative stress, it might serve as a candidate biomarker to study oxidative stress in lung cancer risk. Few epidemiologic studies that have examined the relationship between mtCN in peripheral blood leukocytes (PBL) and lung cancer risk have reached inconsistent conclusions [13–15]. In this study, we examined the relationship between pre-diagnosis leukocyte mtDNA copy number and the risk of lung cancer in a case-control study nested within two prospective cohort studies: the Nurses’ Health Study (NHS) and the Health Professionals Follow-Up Study (HPFS).

## RESULTS

We included 463 case-control pairs from the NHS (285 cases and 285 controls) and the HPFS (178 cases and 178 controls) in this study. Descriptive characteristics of the study populations are provided in Table 1. In both NHS and HPFS, cases were similar to controls with respect to age, alcohol consumption, diabetes status, healthy eating index score (a score describing dietary intake) and physical activity. In the HPFS, cases had a lower body mass index (BMI) than controls.

**Table 1:** Baseline characteristics of lung cancer cases and individually matched controls^a^

	NHS		HPFS	
	Cases (*n* = 285) Mean (SD)	Controls (*n* = 285) Mean (SD)	Cases (*n* = 178) Mean (SD)	Controls (*n* = 178) Mean (SD)
Age at baseline (years)	59.8 (6.2)	59.5 (6.1)	66.0 (7.7)	65.7 (7.7)
Healthy eating index score^b^	33.2 (9.7)	34.3 (10.2)	36.6 (9.2)	37.0 (9.1)
Alcohol consumption (gm/day)	7.7 (12.9)	8.0 (12.5)	15.5 (18.7)	16.2 (20.5)
Type II Diabetes, *n* (%)	9 (3.2)	5 (1.8)	10 (5.6)	12 (6.8)
BMI (kg/m^2^)				
< 25, *n* (%)	171 (60.9)	161 (57.3)	63 (42.9)	47 (31.8)
25 ≤ BMI < 30, *n* (%)	81 (28.8)	85 (30.2)	68 (46.3)	71 (48.0)
≥ 30, *n* (%)	29 (10.3)	35 (12.5)	16 (10.9)	30 (20.3)
Physical activity (METs-hours/week)^c^				
MET < 3, *n* (%)	71 (25.7)	81 (29.2)	24 (13.5)	29 (16.4)
3 ≤ MET < 9, *n* (%)	81 (29.3)	66 (23.8)	29 (16.3)	18 (10.2)
9 ≤ MET < 18, *n* (%)	52 (18.8)	57 (20.6)	33 (18.5)	16 (9.0)
18 ≤ MET < 27, *n* (%)	35 (12.7)	29 (10.5)	14 (7.9)	28 (15.8)
27 ≤ MET, *n* (%)	37 (13.4)	44 (15.9)	78 (43.8)	86 (48.6)

a Samples are matched for smoking status and pack-years of smoking for current/past smokers.

b Healthy eating index score summarized higher intakes of fruit, vegetable, cereal fiber and nuts, higher ratios of chicken plus fish to red meat and polyunsaturated to saturated fat, lower intake of trans-fat^27^.

c MET denotes metabolic equivalent. Met -hours = sum of the average time/week in each activity x MET value of each activity. One MET, the energy spent sitting quietly, is equal to 3.5 ml of oxygen uptake per kilograms of body weight per minute for a 70-kg adult.

We examined mtCN level according to smoking status and found a suggestive inverse association among controls (*P* for trend = 0.07). Compared with never smokers, current heavy smokers (> 24 cigarettes/day) had significantly lower mtCN (*P* = 0.05), as shown in Table 2.

**Table 2:** Estimated least-squares mean log_mtCN and 95% confidence intervals by smoking status among controls

	Log_mtCN (95% CI)	*P* value	Log_mtCN (95% CI)	*P* value	Log_mtCN (95% CI)	*P* value	Log_mtCN (95% CI)	*P* value	*P* for trend
	Never (*n* = 103)		Past (*n* = 426)		Current light smokers (*n* = 234)		Current heavy smokers (*n* = 148)		
Model 1^a^	0.19 (0.07–0.31)	Ref	0.17 (0.10–0.23)	0.73	0.20 (0.12–0.29)	0.86	0.02 (−0.10–0.13)	0.04	0.10
Model 2^b^	0.20 (0.06–0.33)	Ref	0.18 (0.10–0.25)	0.80	0.19 (0.10–0.28)	0.96	0.02 (−0.11–0.14)	0.05	0.07

a Model 1: adjusted for age at blood draw and gender.

b Model 2: adjusted for age at blood draw, gender, BMI, physical activity, alcohol consumption and healthy eating index score.

Our examination did not reveal any overall association between mtCN and lung cancer risk (Table 3). Compared to the high-log_mtCN group, the risk of lung cancer was 1.29 (95% CI = 0.89–1.87) for the median-log_mtCN group, and 1.11 (95% CI = 0.75–1.64) for the low-log_mtCN group. No gender difference was detected (data not shown). In the stratified analysis by smoking status, we found that among current smokers, compared to participants with high levels of log_mtCN, those with median levels of log_mtCN had a significantly higher risk of lung cancer (OR = 2.09; 95% CI = 1.12–3.90), but not those with low levels (OR = 1.37; 95% CI = 0.75–2.48) (Table 4). The interaction between mtCN and smoking status on lung cancer risk was not significant (*P* for interaction = 0.45), possibly due to the fact that we had low power to detect interactions on nominal categories (degree of freedom = 4). We did not find any significant associations among either past or never smokers (Table 4).

**Table 3:** MtDNA copy number and risk of lung cancer

Log_mtCN	Cases (*n* = 463)	Controls (*n* = 463)	OR (95% CI)^a^	OR (95% CI)^b^
Low	151	151	1.19 (0.83–1.71)	1.11 (0.75–1.64)
Median	170	155	1.33 (0.95–1.87)	1.29 (0.89–1.87)
High	141	154	1.00 (Ref)	1.00 (Ref)
*P* for trend			0.56	0.62

a ORs and 95% CIs determined by conditional logistic regression.

b Rs and 95% CIs determined by conditional logistic regression, adjusted for BMI, physical activity, alcohol consumption and healthy eating index score.

**Table 4:** MtDNA copy number and risk of lung cancer-stratified by smoking status

	Current (*n* = 372)		Past (*n* = 412)		Never (*n* = 98)	
Log_mtCN	OR (95% CI)^a^	OR (95% CI)^b^	OR (95% CI)^a^	OR (95% CI)^b^	OR (95% CI)^a^	OR (95% CI)^b^
Low	1.54 (0.88–2.70)	1.37 (0.75–2.48)	1.07 (0.62–1.83)	0.99 (0.53–1.84)	0.83 (0.24–2.91)	0.70 (0.18–2.78)
Median	2.01 (1.13–3.58)	2.09 (1.12–3.90)	1.13 (0.70–1.81)	0.98 (0.57–1.68)	0.75 (0.26–2.15)	0.68 (0.22–2.12)
High	1.00 (Ref)	1.00 (Ref)	1.00 (Ref)	1.00 (Ref)	1.00 (Ref)	1.00 (Ref)
*P* for trend	0.13	0.23	0.59	0.59	0.78	0.68

a OR and 95% CIs determined by conditional logistic regression.

b OR and 95% CIs determined by conditional logistic regression, adjusted for BMI, physical activity, alcohol consumption and healthy eating index score.

To determine whether undiagnosed lung cancer cases at the time of blood collection might have influenced the association, we excluded cases diagnosed within 2 years of follow-up after the blood collection. The results were similar to those with all the cases.

## DISCUSSION

Our data from this nested case-control study suggest that smoking might be associated with less mtCN in PBL, especially among heavy smokers. In addition, although no marginal association was found between mtCN and lung cancer risk, among current smokers the median level of mtCN may be associated with a higher risk of lung cancer than the high level. Such an association was not found in past or never smokers. Further verification with larger sample size as well as biological studies are needed to confirm the association, which if true may indicate a threshold effect in smoking-related and mitochondrial-mediated oxidative stress defense mechanisms. Due to the modest sample size, caution needs to be exercised in interpreting these results.

Smokers had higher levels of oxidative stress which can be detected by plasma lipid peroxidation and urinary secretion of 8-OHdG [11, 12]. In smokers, the correlation between cumulative smoking history and both leukocytosis and elevation of acute-phase reactants reflected a smoking-induced inflammatory response [17]. In addition, smoking causes an increase in oxidative metabolism of macrophages and neutrophils accompanied by increased generation of ROS [18]. Thus, it is conceivable that current heavy smokers have a higher level of oxidative stress and a lower level of mtCN compared to non-smokers.

Studies investigating the role of mtDNA in cancer risk have been accumulating in recent years. Several pathways were found to plausibly link the decreased mtCN with malignant disease development. Altered mitochondrial respiratory function caused by decreased mtCN might lead to deficiency in oxidative phosphorylation, which is the main source of energy production in normal cells. In response, cells increase the generation of adenosine triphosphate by glycolysis, which is often associated with cancer [20, 21]. MtDNA depletion was also found to increase cancer cells’ resistance to apoptosis and lead to epithelial-mesenchymal transition, which are both common in tumor formation and metastatic progression [22, 23]. In our study, the median level of mtCN was associated with two-fold lung cancer risk compared to the high level in current smokers, even though we did not observe any change in lung cancer risk for the low level group.

A case-control study using small sample size carried out in Xuanwei, China and a nested case-control study conducted within the Alpha-Tocopherol, Beta-Carotene (ATBC) Cancer Prevention Cohort Study were the only two studies that have directly measured mtCN and investigated its association with lung cancer risk [13, 14]. Both studies showed that higher mtDNA copy number was associated with higher risk of lung cancer after adjusting for smoking and other covariates. However, the retrospective design in the Xuanwei study and the evaluation of mtDNA content in post-diagnostic biospecimens are prone to reverse causation. Moreover, in a pooled follow-up study where the nested case-control study within ATBC was combined with two additional prospective investigations nested in the Prostate, Lung, Colorectal, and Ovarian (PLCO) cancer screening trial and the Shanghai Women's Health Study (SWHS), mtCN was not consistently associated with lung cancer risk across three prospective study populations from Europe, Asia, and the United States [15]. Similar to our results, mtDNA was found to be inversely associated with lung cancer risk among male smokers in the Prostate, Lung, Colorectal, and Ovarian (PLCO) cancer screening trial [15].

In conclusion, our results suggest that smoking may be associated with decreased mtCN, and that a moderate decrease in mtCN may be associated with development of lung cancer among current smokers. Major strengths of our study are its prospective nature, the elimination of reverse causation by ruling out cases that occurred within the first two years of follow-up, and the stringent control of confounding created by matching cases and controls on smoking status and pack-years of smoking. Additional larger prospective studies with multiple mtCN measurement at different time points are warranted to confirm these associations.

## MATERIALS AND METHODS

### Study populations

Detailed descriptions of the NHS and the HPFS have been previously published [25]. In brief, the NHS began in 1976, when 121,700 registered nurses aged 30–50 years completed baseline questionnaires regarding risk factors for cancers and cardiovascular diseases. Participants completed self-administered mailed follow-up questionnaires biennially with updated information on their lifestyle, medical history, and diet. The HPFS began in 1986 when 51,529 US male health professionals aged 40–75 years completed baseline questionnaires on lifestyle, diet, and medical conditions. The information was updated biennially with follow-up questionnaires. Between 1989 and 1990, blood samples were collected from 32,826 members of the NHS. Between 1993 and 1994, 18,159 HPFS participants provided blood samples.

### Lung cancer case control ascertainment and validation

Lung cancer diagnosis was reported by the participants followed by confirmation through medical records and pathology reports in both NHS and HPFS, or identified through death certificates. Cases were classified as confirmed only if a pathology report indicated that the lesion was a primary lung tumor. All the cases were incident cases after the blood collection.

One control per case was selected from participants who provided blood samples and were free of diagnosed cancer (excluding non-melanoma skin cancer) up to and including the questionnaire cycle in which the case was diagnosed. Controls were matched to cases by age (± 1 year), race, and smoking status at blood collection (never, past, and current). Within the current smokers, controls were further matched on pack-years of smoking (± 1). Within the former smokers, controls were further matched on pack-years of smoking (± 1) before quitting.

### Assessment of smoking history

Smoking information was prospectively collected from both cohort studies. In the NHS, the repeated measurements on cigarette smoking behaviors were obtained via prospective biennial questionnaires. On the initial 1976 questionnaire, participants reported whether they currently smoked, had ever smoked, and the age at which they started smoking. Current smokers reported the number of cigarettes smoked per day, and former smokers reported the age at which they stopped smoking and the number of cigarettes smoked per day before quitting. On each subsequent biennial questionnaire, participants reported whether they currently smoked cigarettes, and at the start of each 2-year follow-up cycle, they were reclassified by smoking status (never, past, or current), quantity of cigarettes smoked and duration among current smokers, and time since quitting among former smokers. The smoking information collected from the HPFS questionnaire was similar to that from the NHS questionnaire.

### mtDNA copy number ascertainment and validation

For quantitative PCR (qPCR)-based assay of relative mtDNA copy number, total DNA was extracted from buffy-coat fractions using the QIAmp (Qiagen, Chatsworth, CA) 96-spin blood protocol. DNA concentrations were determined via pico-green quantitation using a Molecular Devices 96-well spectrophotometer. Relative mtDNA copy number was assessed using a qPCR-based method in a high-throughput 384-well format with an Applied Biosystems 7900HT Real-Time PCR system. DNA concentration was standardized to 5 ng/μL. Ten nanograms of genomic DNA per reaction was added to a 384-well reaction plate and dried down. DNA was reconstituted in 10 μL of multiplex ND2 (single-copy mitochondrial gene) and AluYb8 (nuclear repeat element) PCR reaction mixture. A 20 x multiplex reaction mixture consisted of 18 μM of each of the ND2-forward primer (5′- tgttggttatacccttcccgtacta -3′), ND2-reverse primer (5′- cctgcaaagatggtagagtagatga -3′), AluYb8-forward primer (5′-cttgcagtgagccgagatt -3′), AluYb8-reverse primer (5′- gagacggagtctcgctctgtc -3′), and 5 μM each of the ‘actgcagtccgcagtccggcct’ probe with VIC on the 5′ end and MGBNFQ on the 3′ end, ‘ccctggcccaaccc’ with 6FAM on the 5′ end and MGBNFQ on the 3′ end, plus 20 x multiplex Taqman genotyping mastermix (Taqman). The multiplex reaction thermal cycling profile proceeded as follows: 95°C for 10 minutes, then 30 cycles of 95°C for 15 seconds and 60°C for 1 minute. Triplicate reactions of multiplex reactions were performed on each sample on different plates. The average inter-plate coefficients of variation (CVs) for the ND2 and AluYb8 Ct values were 0.40% and 0.79% respectively among the quality control samples. The average intra-plate CVs were 0.50% and 0.90%. The average slope of the standard curves for both reactions was between −3.5+/−0.3. The R^2^ coefficient of determination was 0.97 or higher for each reaction. The Ct value for each reaction represents the number of PCR cycles required to detect a signal over background fluorescence and is inversely proportional to the amount of DNA. The qPCR-based assay determined the mitochondrial ND2 gene copy number to genomic single-copy gene copy number (N/S) ratio, a value proportional to the average number of mitochondrial DNA copy number. The N/S ratio (−dCt) for each sample was calculated by subtracting the average AluYb8 Ct value from the average ND2 Ct value. The 10 ng DNA standard curve point included on every 384- well plate was used as calibrator DNA to help adjust for inter-assay variability. The relative N/S ratio (−ddCt) was calculated by subtracting the N/S ratio of the calibrator DNA from the N/S ratio of each sample.

### Quality control procedures

In addition to the samples, each 384-well plate contained a 6-point standard curve from 0.625 ng to 20 ng using pooled buffy coat-derived DNA. The purpose of the standard curve was to assess and compensate for inter-plate variation in PCR efficiency. To assess inter-plate and intra-plate variability of threshold cycle (Ct) values, 10% replicate quality control (QC) samples were included in the dataset. The quality control procedures were performed within case-control sets in cohorts separately. CV for repeated sample was 7% while within-person stability within one year showed a Spearman correlation of 0.4, and ICC of 0.29.

### Statistical analysis

Log-transformed mtDNA copy numbers (log_mtCN) were divided into three categories (low, median, and high) based on the median in controls of these categories in the NHS and the HPFS. The adjusted least-squares means of log_mtCN by smoking status were estimated by generalized linear models. Conditional logistic regression was used to estimate the odds ratio (OR) and 95% confidence intervals (CI). Lung cancer risk was examined in a stratified analysis by smoking status (i.e., current, past, and never smokers). All analyses in this study were adjusted for potential confounding factors and performed using SAS (Cary, NC).
